# Lateral movements of a massive tail influence gecko locomotion: an integrative study comparing tail restriction and autotomy

**DOI:** 10.1038/s41598-017-11484-7

**Published:** 2017-09-07

**Authors:** Kevin Jagnandan, Timothy E. Higham

**Affiliations:** 1 Schmid College of Science and Technology, Chapman University, Orange, CA 92866 USA; 2 Department of Evolution, Ecology, and Organismal Biology, University of California, Riverside, CA 92521 USA

## Abstract

Tails are an intricate component of the locomotor system for many vertebrates. Leopard geckos (*Eublepharis macularius*) possess a large tail that is laterally undulated during steady locomotion. However, the tail is readily shed via autotomy, resulting in the loss of tail function, loss in body mass, and a cranial shift in the center of mass. To elucidate the function of tail undulations, we investigated changes in limb kinematics after manipulating the tail artificially by restricting tail undulations and naturally by removing the tail via autotomy. Restricting tail undulations resulted in kinematic adjustments similar to those that occur following tail autotomy, characterized by more flexed hind limb joints. These data suggest that effects of autotomy on locomotion may be linked to the loss of tail movements rather than the loss of mass or a shift in center of mass. We also provide empirical support for the link between lateral tail undulations and step length through the rotation of the pelvic girdle and retraction of the femur. Restriction and autotomy of the tail limits pelvic rotation, which reduces femur retraction and decreases step length. Our findings demonstrate a functional role for tail undulations in geckos, which likely applies to other terrestrial vertebrates.

## Introduction

A defining feature of chordates is the post-anal tail, which has evolved many key functions across taxa^1^. These include courtship^2^, signaling^3, 4^, the maintenance of fat stores^5, 6^, and defense/combat^7, 8^. Tails also have functional roles in animal locomotion, most notably when used directly for propulsion, as in countless swimming animals^9, 10^ and when used to power pentapedal locomotion in kangaroos^11^. Perhaps less obvious is the tail’s role in maintaining balance and enhancing maneuverability or stability^12–16^. Although prehensile tails serve as an extra limb to reduce the risk of falling in arboreal environments^17, 18^, several taxa utilize non-prehensile tails for a similar advantage. Mice have been documented undulating the tail for balance when crossing a narrow perch^19^. Primates with long tails utilize sweeping movements of the tail when navigating narrow supports to alter the momentum of their body^20^, and cats utilize tail adjustments to realign their hips over a perch to avoid falling^21^. Even on broad level terrain, tails can adjust the balance of the body to counteract pitching effects of leg movements^22^, and tails have been shown to be useful for initiating turns and maneuvering^23^.

Lizards are ideal for studying tail function because all of the functions described above are represented within their tremendous diversity. The tail can be dragged behind the lizard, pushed against the substrate during climbing, raised, curled, used as a prehensile “fifth limb”, used for counter-rotation during jumping, or undulated as they walk, run, and/or climb^8, 24–26^. Despite the importance of the tail in various forms of locomotion^15, 27–31^, most lizard species voluntarily shed the tail (autotomy) as a predator-escape strategy^8, 32^. How tail autotomy impacts locomotion has thus become a topic of much interest in recent years^15, 33–35^. Performance effects are variable across species, likely due to differences in the role of the tail in locomotion^25^. Species for which locomotor performance is improved after autotomy generally have large fatty tails that impede faster running^36^, while locomotion is impaired by tail loss in species that depend on the tail for balance, stability, and/or maneuverability^29, 30^.

In some species, autotomy does not influence performance, but significant changes in locomotor mechanics occur. Changes in locomotor kinematics and hind limb ground-reaction forces were recently investigated in the leopard gecko, *Eublepharis macularius* ^37^, a padless desert-dwelling species and an established system for tail autotomy and regeneration^38–42^. Geckos lower their center of mass by taking a more sprawled posture after autotomy, a change that was attributed to a reduction in stability due to the significant loss of caudal mass and cranial shift of the center of mass (*E. macularius* have one of the largest tails relative to body size among lizards)^37^. However, it is unclear if stability is impaired by the change in mass or the loss of tail function. The tail of *E. macularius* serves a primary role in the storage of fats^41^, but unlike many other large-tailed reptiles, the tail is not dragged behind the animal as it walks. Instead, the tail is lifted off the ground and swings laterally. Undulations of the vertebral column generate a standing wave in the trunk that transforms into a traveling wave moving caudally along the tail as the lizard walks^43^.

The function of lateral undulations of the tail during locomotion remains unclear, although several hypotheses have been presented. Tail movements in arboreal mammals are suggested to aid in balance and stability when traversing narrow perches^19–21^. Recent data on green anoles demonstrate that mediolateral tail movements are most prominent on the narrowest perches and compensate for instabilities imposed by a small perch diameter^15^. Undulating the tail during otherwise steady locomotion may also be a useful mechanism for rapidly responding to unexpected perturbations by imparting angular momentum on the body and resisting the destabilizing motion^20, 21, 31, 44^. The tail may also play a role in force generation by the caudofemoralis, the muscle that retracts the femur^28, 45–50^. Undulating the tail could alternately lengthen the caudofemoralis muscles attached to each hind limb as the tail is swung from side to side. Lengthening the muscle to a more optimal length would lead to greater actin-myosin overlap within the muscle sarcomere, which could thus enhance the force generated for propulsion by the caudofemoralis. Tail undulations could also contribute to rotation at the pelvic girdle due to inertial effects. A large undulating tail could provide the momentum necessary for rotating the pelvic girdle in the yaw axis, which could influence both the length of a hind limb step as well as the angle at which the femur can retract to drive propulsion.

**Figure 1:**
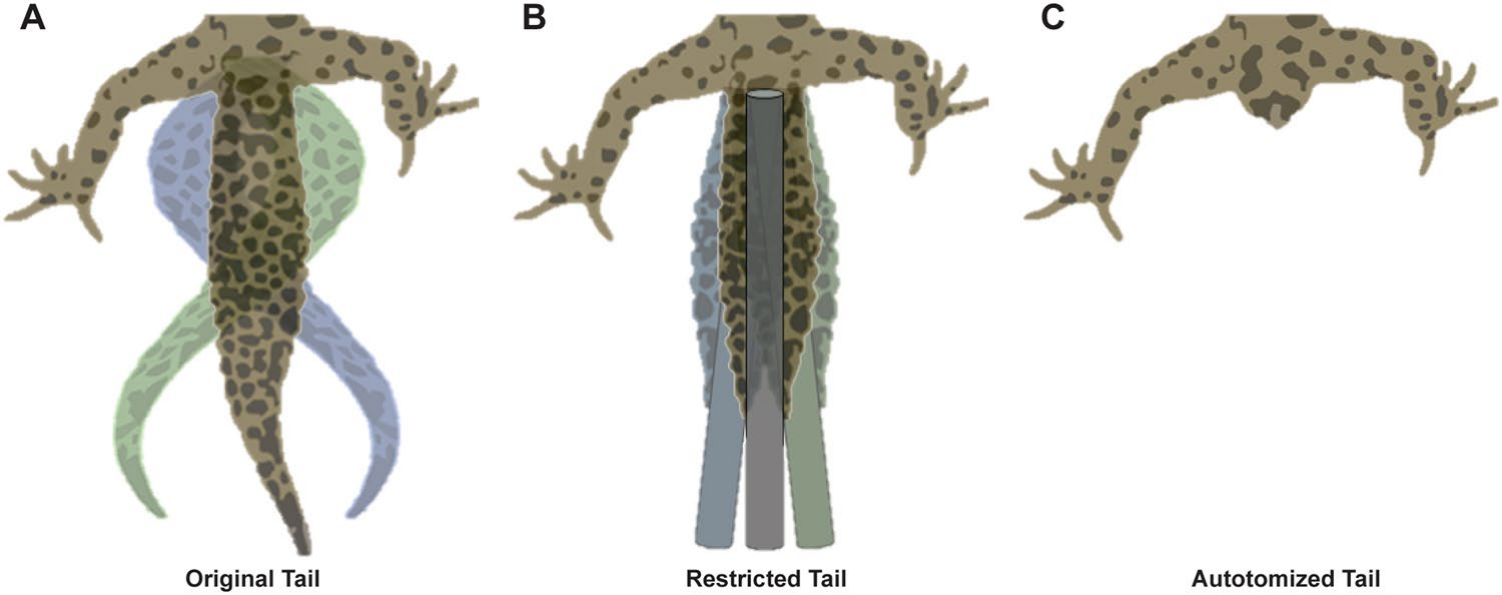
Tail movements under each experimental treatment. Lateral tail undulations freely occur with original tails intact (**A**), while tails are reduced to a stiff rod when restricted with limited movement in the yaw axis (**B**). Tail movement is non-existent after autotomy (**C**).

For both axial and appendicular structures that move during locomotion, function can be revealed by either removing all or some of the structure^37, 51^, by adding to the structure^12, 52^, or by restricting motion of the structure^53, 54^. Although the voluntary loss of the tail has been studied, little is known about the differential role of mass *versus* motion of the tail during locomotion. We examined how the tail is used in leopard geckos walking on level terrain and determined how these tail movements change with speed. We then disabled normal tail movements, both artificially by restricting tail undulations with a graphite rod and naturally by autotomizing the tail in the same individuals (Fig. 1). We hypothesized that restricting the motions of the tail will cause changes in locomotion that are comparable to those that occur following tail autotomy^37^. Thus, we predicted that autotomy-induced changes in locomotion result from the loss of tail undulations, not a loss of mass. We specifically investigated changes in limb joint angles that may augment balance or stability, as well as changes in the rotation of the pelvic girdle when an undulating tail is compromised.

## Results

**Figure 2:**
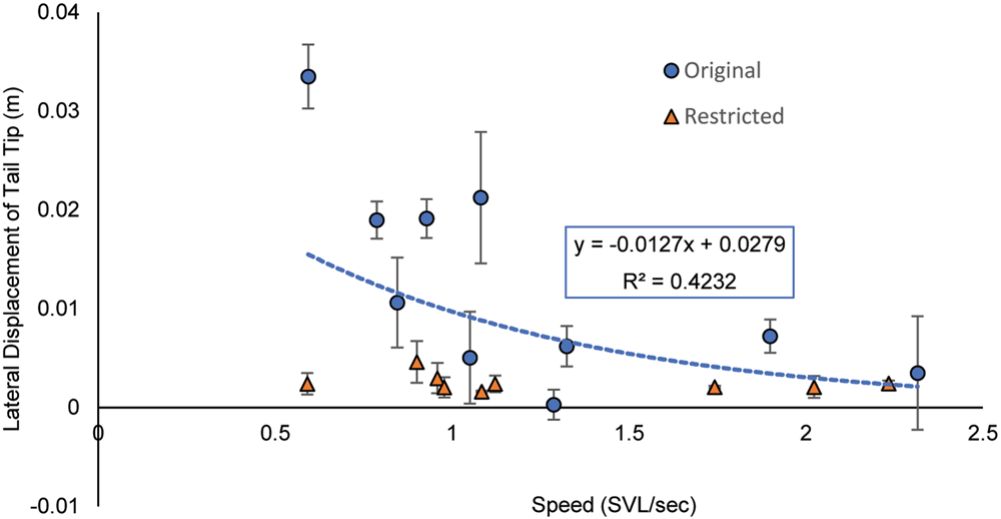
Relationships of lateral displacement of the tail tip with speed. Lateral displacement is measured as the lateral distance of the tail tip relative to the pelvic girdle, as measured on the left side of the body. Data points are means for each individual. Error bars are s.e.m. Regression analysis demonstrates a significant negative relationship of lateral displacement of the tail tip with speed when the tail is unaltered (*P* = 0.042). Lateral displacement of the tail is significantly reduced when the tail is restricted, with no relationship to speed.

In running trials, geckos ran at speeds ranging from 0.59 to 3.36 SVL s^−1^, which was not significantly affected by restricting or autotomizing the tail (repeated measures ANOVA, *F*_2,8_ = 4.075, *P* = 0.060). Lateral displacement of the tip of the tail relative to the pelvic girdle exhibited a significant negative relationship with speed (*F*_1,8_ = 5.870, *P* = 0.042, *R* ^2^ = 0.423) (Fig. 2), although no relationship was observed between the height of the tail and speed (*F*_1,8_ = 0.100, *P* = 0.759, *R* ^2^ = 0.012). Restricting the tail reduced the lateral displacement of the tail as intended (*t* = 3.112, d.f. = 9, *P* = 0.012) and did not affect the tail height off the ground (*t* = 0.734. d.f. = 9, *P* = 0.482).

**Figure 3:**
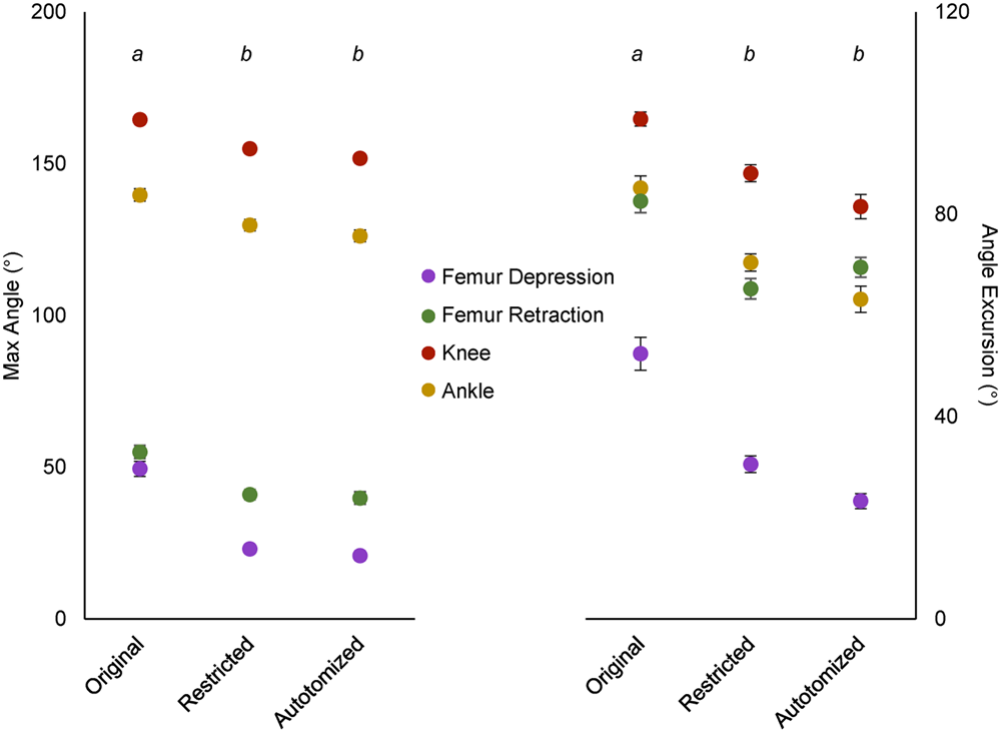
Means of maximum angles (left) and angular excursions (right) of hind limb joints during stance phase. Values for femur depression, femur retraction, knee angle, and ankle angle are means + residuals from ten individuals. Error bars are s.e.m. Letters above each treatment indicate significant differences (repeated measures ANOVA and post-hoc tests for multiple comparisons, *P* < 0.05).

Stride lengths, stance times, and duty factors of the fore- and hind limbs were not significantly impacted by restricting or autotomizing the tail (Table 1). Forelimb joint kinematics were also unaffected. However, step length (the distance traveled during the stance phase of the hind limb) was significantly reduced by restricting (*t* = 3.509, d.f. = 9, *P* = 0.007) and autotomizing (*t* = 3.447, d.f. = 9, *P* = 0.007) the tail. Both restricting and autotomizing the tail also significantly decreased the maximum angles of femur depression (restriction, *t* = 6.225, d.f. = 9, *P* < 0.000; autotomy, *t* = 7.869, d.f. = 9, *P* < 0.000), femur retraction (restriction, *t* = 2.94, d.f. = 9, *P* = 0.016; autotomy, *t* = 3.305, d.f. = 9, *P* = 0.009), knee flexion (restriction, *t* = 4.541, d.f. = 9, *P* = 0.001; autotomy, *t* = 4.627, d.f. = 9, *P* = 0.001), and ankle flexion (restriction, *t* = 3.997, d.f. = 9, *P* = 0.003; autotomy, *t* = 4.157, d.f. = 9, *P* = 0.002) in the hind limbs (Fig. 3). The angular excursion at each of these hind limb joints was also significantly reduced after tail restriction (femur depression, *t* = 3.069, d.f. = 9, *P* = 0.013; femur retraction, *t* = 3.527, d.f. = 9, *P* = 0.006; knee, *t* = 2.939, d.f. = 9, *P* = 0.017; ankle, *t* = 3.577, d.f. = 9, *P* = 0.006) and after tail autotomy (femur depression, *t* = 5.090, d.f. = 9, *P* = 0.001; femur retraction, *t* = 3.115, d.f. = 9, *P* = 0.012; knee, *t* = 5.661, d.f. = 9, *P* < 0.000; ankle, *t* = 5.825, d.f. = 9, *P* < 0.000). No significant differences were observed between the restricted and autotomized tail treatment groups (step length, *t* = 0.859, d.f. = 9, *P* = 0.412; maximum femur depression angle, *t* = 1.089, d.f. = 9, *P* = 0.305; maximum femur retraction angle, *t* = 0.051, d.f. = 9, *P* = 0.960; maximum knee angle, *t* = 1.229, d.f. = 9, *P* = 0.250; maximum ankle angle, *t* = 1.176, d.f. = 9, *P* = 0.270; angular excursion of femur depression, *t* = 2.510, d.f. = 9, *P* = 0.063; angular excursion of femur retraction, *t* = −1.157, d.f. = 9, *P* = 0.277; angular excursion of the knee, *t* = 2.123, d.f. = 9, *P* = 0.063; angular excursion of the ankle, *t* = 1.917, d.f. = 9, *P* = 0.087).

**Table 1:** Summary of kinematic variables in the leopard gecko *Eublepharis macularius* across tail treatments.

Variable	Original	Restricted	Autotomized	*F*-ratio	*P*
**Forelimb**					
Stride length (SVL)*	0.63 ± 0.15	0.60 ± 0.05	0.49 ± 0.06	2.986	0.108
Step length (SVL)*	0.04 ± 0.00	0.05 ± 0.00	0.04 ± 0.00	1.452	0.290
Stance time (s)*	0.54 ± 0.03	0.55 ± 0.03	0.50 ± 0.02	0.640	0.552
Duty factor*	0.70 ± 0.01	0.72 ± 0.01	0.72 ± 0.01	0.765	0.497
**Humerus depression (deg)**					
Maximum	45.59 ± 8.31	34.29 ± 4.88	29.30 ± 2.91	0.898	0.445
Angular excursion	101.38 ± 15.88	72.67 ± 7.72	72.26 ± 4.30	1.818	0.223
**Humerus retraction (deg)**					
Maximum	54.78 ± 2.68	68.51 ± 2.45	73.02 ± 3.13	2.897	0.113
Angular excursion	44.41 ± 1.80	51.21 ± 2.57	59.23 ± 2.41	3.505	0.081
**Elbow angle (deg)**					
Maximum	151.04 ± 1.23	144.82 ± 1.91	142.56 ± 1.59	1.986	0.199
Angular excursion	92.49 ± 2.27	83.17 ± 2.64	93.76 ± 2.73	4.383	0.052
**Wrist angle (deg)**					
Maximum	165.31 ± 2.00	161.83 ± 1.43	164.83 ± 1.45	1.233	0.341
Angular excursion	71.93 ± 3.09	70.38 ± 2.51	73.33 ± 2.73	0.353	0.713
**Hind limb**					
Stride length (SVL)*	0.62 ± 0.15	0.72 ± 0.05	0.70 ± 0.06	1.275	0.331
Step length (SVL)*	0.06 ± 0.00	0.05 ± 0.00	0.05 ± 0.00	5.836	**0.027**
Stance time (s)*	0.64 ± 0.03	0.60 ± 0.03	0.56 ± 0.01	0.923	0.436
Duty factor*	0.78 ± 0.01	0.78 ± 0.01	0.77 ± 0.01	0.578	0.583
**Femur depression (deg)**					
Maximum	49.44 ± 2.49	23.01 ± 1.44	20.79 ± 1.30	29.601	<**0.000**
Angular excursion	52.42 ± 3.25	30.56 ± 1.63	23.29 ± 1.48	30.447	<**0.000**
**Femur retraction (deg)**					
Maximum	55.00 ± 2.18	40.94 ± 1.65	39.82 ± 2.03	6.106	**0.025**
Angular excursion	82.57 ± 2.29	65.26 ± 2.03	69.50 ± 1.95	5.637	**0.030**
**Knee angle (deg)**					
Maximum*	164.49 ± 0.93	154.84 ± 1.47	151.75 ± 1.30	1.674	**0.003**
Angular excursion*	98.83 ± 1.39	88.08 ± 1.68	81.48 ± 2.40	14.282	**0.002**
**Ankle angle (deg)**					
Maximum	139.65 ± 2.03	129.69 ± 1.90	126.19 ± 1.97	9.85	**0.007**
Angular excursion	85.19 ± 2.38	70.41 ± 1.71	63.19 ± 2.61	16.589	**0.001**
Speed (SVL/sec)	1.23 ± 0.16	1.50 ± 0.22	1.85 ± 0.36	4.075	0.060

Means + residuals (±s.e.m.) for each variable are given for original, restricted, and autotomized tail treatments. Statistical significance (repeated measures ANOVA) of changes in each variable is also given. Significant results are indicated in bold type. Asterisks indicate variables that had a significant relationship (α ≤ 0.10) with speed.

**Figure 4:**
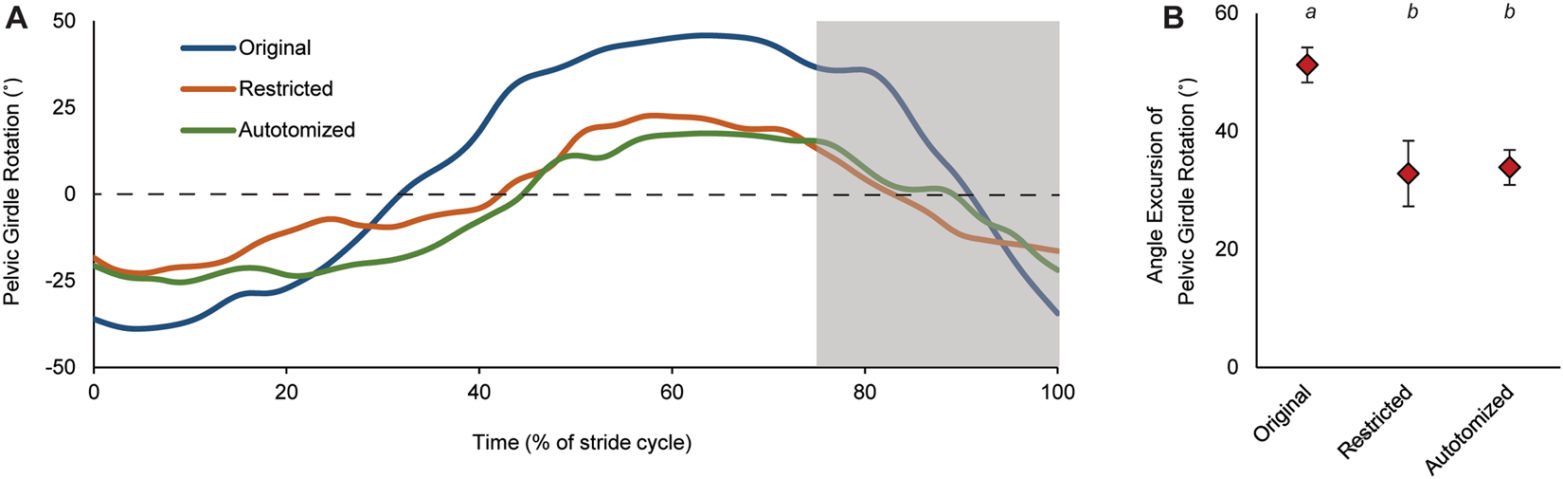
Changes in pelvic girdle rotation with restriction and autotomy. (**A**) Degree of rotation of the pelvic girdle over time (as a percentage of stride cycle) is provided for a representative hind limb stride of a leopard gecko with its original (blue), restricted (orange), and autotomized (green) tail. Negative values indicate that the pelvic girdle is rotated to the right (toward the hind limb being observed) and positive values indicate that the pelvic girdle is rotated to the left (toward the opposite hind limb). The non-shaded region represents the stance phase of the observed hind limb and the area shaded in gray represents the swing phase. (**B**) Means of angular excursion of the pelvic girdle in the yaw axis across treatments from ten individuals. Error bars are s.e.m. Letters above each treatment indicate significant differences (repeated measures ANOVA and post-hoc tests for multiple comparisons, *P* < 0.05).

Pelvic girdle rotation decreased significantly when the tail was compromised, as indicated by a lower angular excursion in lizards with restricted (*t* = 2.287, d.f. = 9, *P* = 0.048) and autotomized (*t* = 3.129, d.f. = 9, *P* = 0.012) tails when compared to lizards with original tails intact (Fig. 4). No significant differences in pelvic girdle rotation were observed between the restricted and autotomized treatments (*t* = −0.247, d.f. = 9, *P* = 0.810).

## Discussion

Tail autotomy in lizards can result in a significant loss of body mass, a cranial shift in the center of mass, and a loss of function that results from tail motion. While the loss of mass and shifted center of mass occur simultaneously and cannot be decoupled, we investigated the functional role of the tail by experimentally restricting its lateral movement. Analysis of locomotor kinematics of *E. macularius* under experimental conditions in which the tail was compromised revealed the function of tail motions when walking and their relationship to pelvic rotation and step length. Specifically, we observed a more sprawled posture when lateral undulations of the tail were restricted and when the tail was completely autotomized, suggesting that geckos must compensate for not only the loss of caudal mass, but also for the loss of tail motion after an autotomy event. Additionally, restricting tail undulations reduced the lateral rotation of the pelvic girdle, retraction of the femur, and step length, thereby providing evidence for a significant role of the tail in gecko locomotion. These results, elaborated below, reveal key functions of tails during locomotion that are likely applicable to any terrestrial vertebrate that relies on tail motion to move effectively.

Despite having a large fatty tail that accounts for one-fourth of the animal’s body mass, the tail of *E. macularius* is slightly raised and laterally undulated instead of being dragged on the ground while walking. As the base of the tail moves laterally, the femora are alternately retracted to generate propulsion. The base of the tail is flexed towards the protracted hind limb during each cycle of hind limb movement, and the remainder of the tail follows this basal movement in an undulatory manner. Interestingly, we found that lateral displacement of the tip of an intact tail exhibits a negative relationship with the speed at which the gecko walks (Fig. 2), suggesting that the tail swings less at higher speeds. This more rigid posture of the tail straightens the profile of the lizard, and is suggested to be appropriate when lizards are moving forward quickly^12^. It is likely that laterally undulating the tail is inefficient at higher speeds given its substantial mass. Accelerating and decelerating the large tail when moving at high speeds would require more force and power due to the reduced amount of time available for swinging the tail from side to side, which might simply not be possible for the geckos.

**Figure 5:**
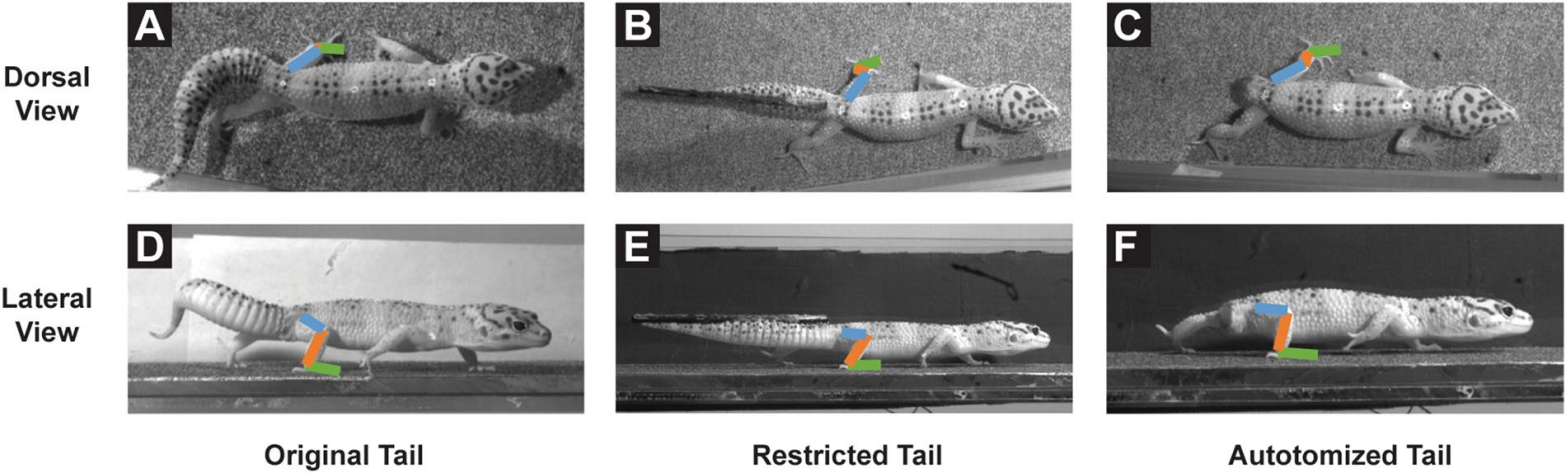
Video frames of leopard geckos under each experimental treatment. Dorsal (**A**–**C**) and lateral (**D**–**F**) are shown for geckos with original (**A**,**D**), restricted (**B**,**E**), and autotomized (**C**,**F**) tails. Colored lines are superimposed over the segments of the observed hind limb to visualize changes in joint angles.

After losing its tail, *E. macularius* adopts a more sprawled posture during locomotion, as previously indicated by decreases in femur depression, femur retraction, knee angle and ankle angle^37^. This locomotor response to autotomy is hypothesized to augment stability and balance that may be impaired due to the altered mass distribution and/or the loss of tail as a stabilizing appendage. Restricting the tail allowed us to tease apart the locomotor effects of autotomy due to altering mass/center of mass versus losing tail function. By effectively modifying the tail into a stiff rod, the gecko was permitted to lift the tail off the ground to prevent friction, but prevented from swinging and laterally undulating the tail as it walked. This modification produced the same locomotor response as autotomizing the tail (Fig. 5). Both removing and restricting the tail can impact the location of the center of mass, with removal shifting the center of mass forward^37, 55^ and restriction limiting lateral displacements of the center of mass^15, 28^. Forelimb kinematics were unaffected by restriction and autotomy, but maximum joint angles and angular excursions in the hind limbs decreased (Fig. 3). These results suggest that tail undulations have a functional role in locomotion on level terrain, a role that is lost after autotomy and requires compensation by altering hind limb kinematics. In fact, it is likely that the impacts of autotomy on locomotion are a result of losing potentially beneficial tail movements, and not necessarily related to the loss of mass.

The function of tail undulations during steady locomotion is more clearly elucidated by the observed changes in pelvic rotation and its downstream effects on femur retraction and step length. Both restricting and autotomizing the tail reduced the degree of rotation of the pelvic girdle throughout the stride (Fig. 4). We hypothesize that swinging the heavy tail laterally provides momentum for rotation at the pelvic girdle via an inertial effect. As the base of the tail is rotated laterally, the length of the tail follows this movement in an undulatory manner. Given the substantial mass of the tail being shifted at the caudal end, the angular momentum of the tail contributes to rotating the pelvic girdle in the yaw axis. Lizards generally exhibit greater pelvic rotation in order to facilitate a more sprawled posture compared to most other terrestrial quadrupeds^56–59^. Thus, a reduction in pelvic rotation should be expected to generate a more upright posture. This is in stark contrast to what is observed after tail autotomy, in which lizards become more sprawled to maintain stability^37^. Decreased pelvic rotation after autotomy thus results in a reduced step length during steady locomotion to maintain the sprawled posture. Although walking speed was not affected by the observed reduction in step length, we suspect that maximal sprint speed would likely be negatively impacted. We did not assess this in our study as we were mainly interested in the impact of tail autotomy and immobilization on kinematics. Additionally, pelvic rotation influences the angle at which the femur can protract and retract^28, 49, 50^. The reduction in the angle of femur retraction observed in lizards with restricted and autotomized tails (Table 1) coincides with the reduction in pelvic rotation. Our data provide empirical support for the proposed link between lateral tail undulations and step length by rotation of the pelvic girdle and retraction of the femur^60^. Autotomy is therefore likely to impact lizards that have a functional tail that provides momentum for rotating the pelvic girdle.

Our findings demonstrate that the tail serves a functional role in locomotion by undulating and rotating the pelvic girdle, thus contributing to femur retraction and step length. To further reveal the locomotor function of tail undulations in terrestrial lizards, we propose a series of future experiments that will elucidate how the tail is used and how animals compensate for the lost appendage. First, the effects of tail loss on dynamic stability and maneuverability should be tested by examining if/how lizards utilize the tail to navigate obstacles, drops, and turns. Experiments that record the timing and intensity of muscle activation in the tail will reveal whether these movements are passively or actively controlled, providing important insight into how tail undulations are modulated. Passive control may suggest that undulating the tail occurs by simply dissipating energy from the laterally undulating body during locomotion, while active control would suggest neuromuscular input that may be necessary for regulating balance or stability. Electromyography experiments would also be insightful when testing how the tail undulations affect the activation of the caudofemoralis and its role in retracting the femur^45, 46^. Finally, we hope to explore the evolution of tail function by using these methods to explore the diversity of tail morphologies and their related locomotor functions across lizard taxa.

Tail autotomy in lizards provides an effective and natural system for understanding tail function. Hypothesized functions of tails commonly arise from studies on tail autotomy and locomotion. A negative impact of tail loss on performance suggests that the tail serves a role in balance, stability, maneuverability, or propulsion^29, 30, 61^. Other attempts at assessing tail use in locomotion involve invasive surgeries with irreparable effects on the study animals^19, 21, 27^. However, tail autotomy allows for a removal of the tail in a natural manner with minimal physiological effects^62^ in order to study its function.

## Materials and Methods

### Study organisms

Ten adult *E. macularius* (mass, 36.3 ± 1.9 g; SVL, 104.6 ± 2.1 mm) with original tails intact were obtained from commercial suppliers and housed in terraria (50.8 × 25.9 × 2.0 cm) maintained at 28–33 °C. Geckos were fed a diet of live crickets *ad libitum*, but fasted the day before the experiment until trials were complete. Prior to experimental trials, white nail polish was applied to the following points on the animals to visualize body and joint movements in high-speed videos: dorsal midpoint of the body, center of the pectoral/pelvic girdles, shoulder/hip, elbow/knee, wrist/ankle, and the metapodial-phalangeal joint of the middle toe. Joints were marked on the right forelimbs and hind limbs. Five points were also evenly distributed from the base of the tail to the tail tip to track the tail movements. All animal research was conducted in accordance with the University of California, Riverside Animal Care and Use Protocols (A-20110025 and A-20110038) with approval from the Institutional Animal Care and Use Committee (IACUC).

### Experimental set-up

Stride kinematics were obtained from each lizard as it ran on a level trackway (1.0 × 0.13 m) with sandpaper substrate to prevent slipping. A mirror mounted at 45° above the trackway provided a dorsal view for the trials. The temperature of the experimental room was maintained at ~30 °C. Lizards were recorded moving along the trackway under three tail treatments: original, restricted, and autotomized (Fig. 1). After recording trials with a lizard’s original tail intact, a lightweight (<1.0 g) hollow graphite rod was attached along the entire length of the tail using non-toxic glue. The rod restricted undulations of the tail, while still permitting the lizard to lift its tail off the ground to prevent friction drag while walking. Locomotor trials were then repeated with the restricted tail. Following these trials, the rod was gently removed from the tail, and the base of the tail was gently pinched to initiate autotomy at the proximal-most fracture plane. Trials were then repeated for lizards with autotomized tails. Between trials for each treatment, each individual was allotted 20–30 minutes to rest in order to minimize potential effects of fatigue or stress associated with the restriction and removal of the tail^62^. However, we limited the amount of walking between trials to avoid any short-term adjustments.

### Stride kinematics

Locomotor movements were captured at 250 frames s^−1^ with a shutter speed of 1/2000 s using two Photron APX-RS cameras (Photron USA, San Diego, CA, USA), one aimed at the lateral view of the lizard and the other recording a dorsal view from the mirror. Cameras were synchronized with an external trigger. A pre-measured calibration object constructed of LEGO™ blocks was used to generate 3D coordinates for digitizing. Three to five forelimb and hind limb strides were recorded for each individual under each tail treatment, providing a total of at least nine strides per individual. Each stride was representative of an individual moving at a relatively constant speed, at least two strides after the initial acceleration. We digitized the points marked on the animals using DLT DV5 custom software^63^ for MATLAB (version R2012a, The MathWorks, Natick, MA, USA) to obtain *x*, *y*, and *z* coordinates to describe antero-posterior, medio-lateral, and dorso-ventral movements, respectively. These coordinates were then used to calculate speed, stride length, stance time, duty factor, and joint angles for the fore- and hind limb throughout each stride. Details of these calculations are available elsewhere^37, 64^. Tail coordinates were used to calculate the height of the tail off the ground and lateral displacement of the tail (measured as the lateral displacement of the tail tip relative to the pelvic girdle) throughout each stride. Only the movements of the tail in the yaw axis were considered here.

### Statistical analyses

Averages of each kinematic variable for each individual per tail treatment were used for all statistical analyses. For the tail variables (tail height and lateral displacement), a regression analysis was used to examine the relationship between tail movements and walking speed. The effects of speed on fore- and hind limb joint kinematics were removed by regressing the variables against body speed. Residuals of the variables that had a significant relationship (α ≤ 0.10) with speed were used for subsequent statistical analyses, while all other data were analyzed in their original form. A repeated-measures ANOVA was used to compare each variable between original, restricted, and autotomized tail treatments, and *post hoc* tests with Bonferroni corrections were used for pair-wise comparisons among the treatments. Assumptions for normality and equal variances were not violated for any of the variables measured. All statistical analyses were performed using SYSTAT 13.00.05.
